# Long Noncoding RNA LINC00467 Promotes Glioma Progression through Inhibiting P53 Expression via Binding to DNMT1

**DOI:** 10.7150/jca.41942

**Published:** 2020-03-04

**Authors:** Yin Zhang, Xuefeng Jiang, Zhisheng Wu, Daling Hu, Junli Jia, Jinfeng Guo, Tian Tang, Jialin Yao, Hongyi Liu, Huamin Tang

**Affiliations:** 1 Department of Neurosurgery, Sir Run Run Hospital, Nanjing Medical University.; 2 School of Basic Medical Sciences, Nanjing Medical University.; 3 Department of Geriatrics, Sir Run Run Hospital, Nanjing Medical University.; 4 Department of Neurosurgery, The Affiliated Brain Hospital of Nanjing Medical University.

**Keywords:** LINC00467, Glioma, Proliferation, Invasion, DNMT1, P53

## Abstract

**Purpose**: This study aimed to investigate whether long noncoding RNA (lncRNA) LINC00467 could regulate proliferative and invasive abilities of glioma cells via p53 and DNA methyltransferase 1 (DNMT1), so as to participate in the occurrence and progression of glioma. **Methods**: LINC00467 expression in glioma was analyzed by GEPIA database and LINC00467 expression in glioma cell lines was detected by qRT-PCR. The regulatory effects of LINC00467 and p53 on proliferative, invasive capacities and cell cycle were conducted by CCK-8 and EdU assays, transwell assay and flow cytometry, respectively. The binding conditions between LINC00467, DNMT1 and p53 were determined by RNA immunoprecipitation (RIP) and Chromatin immunoprecipitation (ChIP) assays. Western blot was conducted to determine whether LINC00467 could regulate p53 in glioma cells. Finally, rescue experiments were carried out to evaluate whether LINC00467 regulates proliferative and invasive abilities of glioma cells through p53. **Results**: The expression of LINC00467 was significantly up-regulated in tumor samples than that in normal samples, which was not correlated with patient survival time. Besides, expression of LINC00467 was higher in glioma cells than that of negative control cells. Upregulation of LINC00467 promoted proliferative and invasive abilities, and accelerated cell cycle in G0/G1 phase of U87 and LN229 cells. The results of RIP and ChIP assays demonstrated that LINC00467 could bind to DNMT1 and inhibit p53 expression. Overexpression of p53 partially reversed the enhancement of LINC00467 on proliferative and invasive abilities of glioma cells. **Conclusion**: These results indicated that high expression of LINC00467 could promote proliferative and invasive abilities of glioma cells through targeting inhibition of p53 expression by binding to DNMT1.

## Introduction

Glioma represents the most frequent intracranial malignant brain tumors in adults, characterized by rapid growth, early metastasis and high lethality 1,2. Despite great progress on glioma treatment that has been made, the prognosis of glioma still remains grim 3. Mounting researches have been performed to detect the potential mechanisms associated with glioma, only a few have been revealed so far 4, 5. Therefore, further exploring the potential mechanisms of these genomic changes in glioma is urgently required.

Long noncoding RNAs (lncRNAs) are a type of transcript that is longer than 200 nucleotides in length with restricted protein-coding capacity 6-8. It has been reported that the expression levels of lncRNAs were dysregulated in various tumors 9,10. Mounting evidence exhibits that lncRNAs are the key regulators in several cellular and biological processes including cellular proliferation, metastasis, and cell apoptosis. For examples, lncRNA small nucleolar RNA host gene 20 (lncSNHG20) is upregulated and can promote cell proliferation and migration in non-small cell lung cancer 11, lncRNA Growth Arrest Specific 5 (lncGAS5) differentially regulates cell cycle arrest and apoptosis in human neuroblastoma through activating breast cancer type 1 susceptibility protein (BRCA1) and p53 12, H19 can promote tumor growth and indicate a poor prognosis in colorectal cancer 13. Although lncRNAs are regarded as candidate therapeutic targets, the role they play on glioma is little known.

LINC00467 is a novel lncRNA localized at the chromosomal locus 1q32.3, and has been reported to be markedly dysregulated in hepatocellular carcinoma 14, lung adenocarcinoma 15 and neuroblastoma 16. Additionally, it has been confirmed that LINC00467 could promote cell proliferation, migration and invasion of lung adenocarcinoma cells 17. However, the specific mechanism of LINC00467 in glioma remains to be further investigated.

In this study, we discovered that LINC00467 expression was increased in glioma tissues and cell lines, and overexpression of LINC00467 could significantly promote cell proliferation and invasion in vitro. Additionally, flow cytometric analysis indicated that upregulation of LINC00467 promoted cell cycle and inhibited cell apoptosis. Furthermore, we found that LINC00467 could bind to DNMT1 and inhibit the expression of p53. Taken together, all findings suggested that LINC00467/DNMT1/p53 regulatory axis might be involved in the occurrence of glioma.

## Materials and methods

### GEPIA database

GEPIA database (http://GEPIA.cancer-pku.cn/index.html) was used to assess the expression level of LINC00467 and survival prognosis of glioma patients. The database can be used to analyze differential genes in tumors, including gene co-expression analysis, gene OS and DFS survival curve analysis, and gene and clinical stage correlation analysis.

### Cell lines and culture conditions

Human glioma cell lines LN229, LN308, U87, LN229 and human normal glial cell line HEB were purchased from American Type Culture Collection (ATCC, Manassas VA, USA). All cells were cultured in RPMI-1640 medium supplemented with 10% fetal bovine serum (Life Technologies, USA), 100 U/ml penicillin and 100 μg/ml streptomycin (Gibco, NY, USA). Cells were incubated at 37 °C in humidified air containing 5% CO_2_. Cell passage was performed using trypsin until 80%-90% of confluence.

### Cell transfection

Small interference RNAs (siRNAs) or overexpressed plasmid for LINC00467, DNMT1 and p53 and corresponding negative controls were synthesized by GenePharma (Shanghai, China) and transfected into the cells to a final oligonucleotide concentration of 10 nmol/l. Transfection was performed using Lipofectamine 2000 (Invitrogen, Carlsbad, CA, USA) in accordance with the manufacturer's instructions. The cells were tested after 48 h of transfection.

### RNA extraction and quantitative real-time polymerase chain reaction

Total RNA was extracted from cells through using TRIzol reagent (Invitrogen, Carlsbad, CA, USA) in accordance with the manufacture's guide. Then Reverse Transcription Kit (Takara, Tokyo, Japan) was utilized to reversely transcribe RNA into cDNA. Quantitative real-time polymerase chain reaction (qRT-PCR) was carried out to detect the mRNA expression by utilizing SYBR® Premix Ex TaqTM (Takara) on the ABI 7500HT (Applied Biosystems, Foster City, CA, USA) following the protocols. The expression of mRNA was normalized to the Glyceraldehyde‐3‐phosphate dehydrogenase (GAPDH) expression level. Each experiment was replicated thrice, and the relative expression was calculated utilizing the 2^-ΔΔCt^ method. All primers involved are listed in Table 1.

### Cell viability assay

Cell-Counting Kit 8 (CCK8; Dojindo Laboratories, Kumamoto, Japan) reagent was utilized to analyze the proliferative capabilities of U87 and LN229 cells which received different treatments. After treatments, exponentially growing cells (3 × 10^3^ per well) were plated into 96-well culture plates. After cells were cultured for 1 day, 2 days, 3 days, 4 days, 5 days, 6 days, respectively, CCK8 solution (10 μL) was added to each well following the manufacturer's instructions. After the cells were incubated at 37 °C for an additional 1 h, the OD value at 450 nm was recorded utilizing a microplate reader. Each assay was repeated in triplicate.

### Ethynyl deoxyuridine (EdU) assay

Proliferating cells were determined by using the 5-ethynyl-2'-deoxyuridine (EdU) abeling/detection kit (Ribobio, Guangzhou, China) according to the manufacturer's protocol. The transfected LN229 and U87 cells were treated with 50 μmol /L of EdU for 2 hours at 37°C and then the cultured cells were fixed with 4% paraformaldehyde for 30 minutes and stained with 1 × Apollo reaction cocktail for 30 minutes before being incubated with 100 μL of Hoechst33342 at 5 μg /mL for 30 minutes. The percentage of EdU positive cells was examined using a fluorescent microscope. All the assays were repeated for three times.

### Cell invasion assay

For cell invasion assay, transfected cells (1×10^5^) were cultured in the upper invasion chambers (8 um pore size, Millipore Corporation, Billerica, MA) coated with Matrigel RPMI-1640 medium without serum, while 600 μl of medium supplemented with 10% FBS was added to the lower chamber. After 48 hours, the cells which couldn't migrate through the chamber were removed. The cells from the lower chamber were fixed using 4% paraformaldehyde for 30 minutes, and stained using crystal violet for 20 minutes. Penetrating cells were captured in 5 randomly selected fields of each sample. All the assays were conducted three times independently.

### Cell cycle assay

At 48 hours after transfection, U87 and LN229 cells were harvested and washed using ice-cold PBS solution. Subsequently the cells were fixed with 70% ethanol overnight at 4 °C before being re-suspended using propidium iodide (PI)/RNase A solution (5 μg/mL PI and 100 mg/mL RNase A) and incubated for 15 min at room temperature in the dark. Then the flow cytometer (Millipore Guava) was utilized to analyze cell cycle. All the assays were conducted three times independently.

### Cell apoptosis assay

The cells were harvested 48 h after the transfection and washed twice using cold PBS. Then cells were re-suspended in binding buffer (BD Biosciences). Annexin V-FITC was utilized to stain the cells which were then resuspended using binding buffer (100 μL), before 5 mL of allophycocyanin-annexin V (BD Biosciences) and 50 mg/mL propidium iodide (Invitrogen) were added to it. Subsequently the cells were mixed and incubated for 15 min in the dark at room temperature. Flow cytometry (Millipore Guava) was used to detect and quantify the apoptotic cells based on the manufacturer's instructions.

### Dual‐luciferase reporter assay

Briefly, glioma cells were plated in 24-well plates, cultured overnight, and transfected using Lipofectamine 2000 according to the manufacturer's protocol. After transfection for 24 h, Firefly and Renilla luciferase activities were measured using the dual-luciferase reporter assay system (Promega, Madison, WI, USA). The ratios of luminescence from Firefly to Renilla luciferase were normalized through three independent experiments.

### Subcellular fractionation location

Nuclear and cytoplasmic RNA were extracted utilizing the PARIS Kit (Life Technologies, USA) following its manufacturer's guides. Then we performed qRT-RCR to detect relative RNA level isolated from each fraction. U1 acted as nuclear control transcript, while GAPDH functioned as cytoplasmic marker.

### RNA-binding protein immunoprecipitation assay

RNA-binding protein immunoprecipitation (RIP) assay was conducted on U87 and LN229 cells by using EZ-Magna RIP Kit (Millipore, Billerica, MA, USA) following the manufacturer's instructions. The transfected cells were washed with ice-cold PBS and then mixed with an equivalent volume of RIP lysis buffer. Next, the lysis products were incubated with 5 μg of human anti-DNMT1 antibody (Millipore, Billerica, MA, USA) or negative control mouse IgG (Millipore, Billerica, MA, USA) for 2 h at 4 °C. Subsequently, each sample was mixed with 50 μL of prepared magnetic beads and incubated at 4 °C overnight. The beads were briefly washed (five times in total) with RIP buffer and resuspended in 500 μL of TRIzol LS (Life Technology, Carlsbad, CA, USA). Finally, purified RNA was subjected to qRT-PCR analysis.

### Chromatin immunoprecipitation (ChIP) assay

EZ-ChIP kit (Millipore) was utilized to perform ChIP assays following its manufacturer's protocol. The cells were fixed in 1% formaldehyde at room temperature for 10 min. After being washed, the cells were scraped and swelled in hypotonic swelling buffer, and then incubated on ice for 10 min. Subsequently, the nuclei were lysed in SDS lysis buffer. IgG (Santa Cruz) and Antibodies against DNMT1 (Abcam) were used for IP. Real-time PCR was performed to amplify the DNMT1-binding region of the p53 promoters. The PCR primer sequences were: sense: 5′- GGGTAAGTTTTTGATTGAATTTGAT-3′ and antisense: 5′- CAAAACTCCACTCCTCTACCTAAAC-3′.

### Western blot

Aggregate proteins in U87 and LN229 cells were extracted utilizing RIPA buffer (Radio-Immunoprecipitation assay buffer, Beyotime). Moreover, protein concentrations were quantified using BCA Protein Assay Kit Protein (Beyotime, Nantong, China). Equal quantities of samples were separated by utilizing 10 per cent SDS-PAGE gel before being added to the PVDF membrane (Millipore, Boston, MA, USA). Next, the membranes were blocked using the blocking solution for 1 hour and then incubated overnight at 4 °C using primary antibodies against DNMT1, p53 and GAPDH (Abcam, MA, USA). After being washed, corresponding HRP-conjugated secondary antibodies were used for incubation at room temperature for 2 hours. GAPDH was put to serve as an endogenous control. The images of protein bands were captured with the help of a Tanon detection system using ECL reagent (Thermo).

### Statistical analysis

SPSS 22.0 (SPSS, Chicago, IL, USA) and Graphpad Prism 6.0 (GraphPad Software, La Jolla, CA, USA) were utilized to analyze the data. The differences between measured groups were analyzed via the Student t test. Data were presented as the mean ± one standard deviation (SD). P < 0.05 was considered as statistically significant.

## Results

### LINC00467 expression is remarkedly increased in glioma

**Figure 1:**
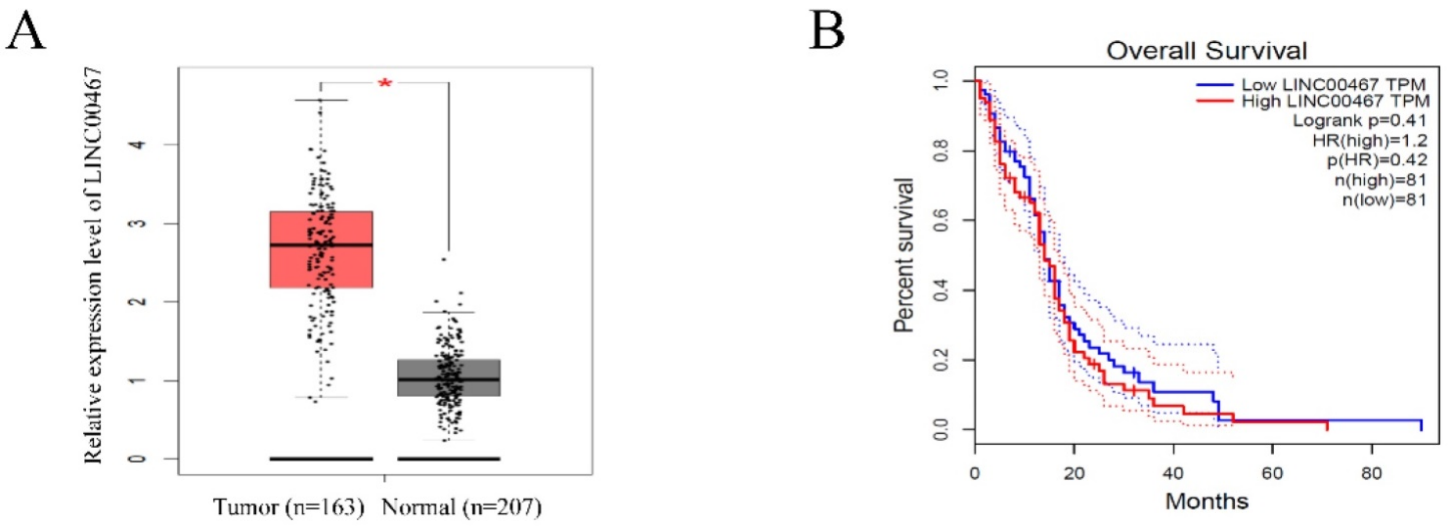
** LINC00467 expression is remarkedly increased in glioma** A. LINC00467 levels in glioma tissues (n=163) and normal tissues (n=207) analyzed in TCGA STAD database. B. The Kaplan‐Meier curve depicts the overall survival of 162 patients with glioma. Error bars indicate mean ± standard errors of the mean. *P < 0.05. TCGA, the Cancer Genome Atlas.

To investigate the expression of LINC00467 in human glioma, we searched the Cancer Genome Atlas (TCGA) database and found that the LINC00467 expression was significantly elevated in glioma tissues compared with normal tissue (Figure 1A). However, the results of TCGA database indicated that the expression level of LINC00467 was not associated with the overall survival rates of glioma patients (Figure 1B). The results suggested that LINC00467 may play a pivotal role in the progression of glioma.

### LINC00467 promoted glioma cell proliferation and invasion

**Figure 2:**
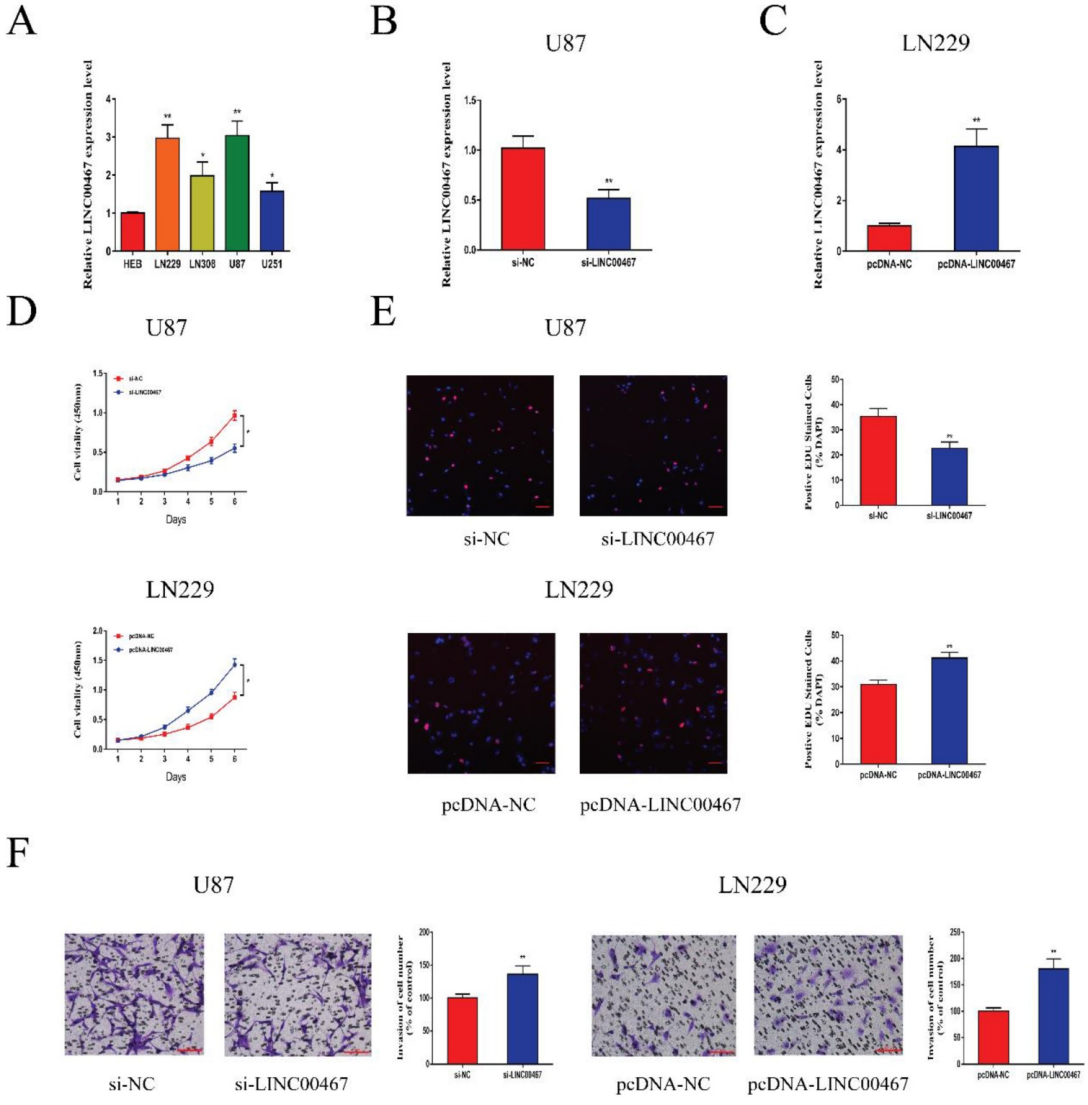
** LINC00467 promoted glioma cell proliferation and invasion** A. Quantitative real-time polymerase chain reaction (qRT-PCR) was used to analyse the expression of LINC00467 in human normal glial cell line (HEB) and glioma cells. B. QRT-PCR analysis of LINC00467 expression in si-NC, si‐LINC00467 in U87 cells. C. QRT-PCR analysis of LINC00467 expression in pcDNA-NC, pcDNA‐LINC00467 in LN229 cells. D. The CCK8 assay was used to determine the viability of si‐LINC00467 transfected or pcDNA‐LINC00467 transfected glioma cells. E. The EdU assay was used to determine the viability of si‐LINC00467 transfected or pcDNA‐LINC00467 transfected glioma cells. F. Downregulation of LINC00467 in U87 cells inhibited cell invasion while overexpression of LINC00467 in LN229 cells promoted cell invasive capability. The data represent the mean ± SEM from three independent experiments. *P < 0.05, **P < 0.01. CCK8, Cell-Counting Kit 8; EdU, Ethynyl deoxyuridine.

The expression levels of LINC00467 in glioma cell lines and negative control cells were detected using qRT-PCR. The results revealed that LINC00467 was upregulated in glioma cells compared with the controls, especially in U87 and LN229 cells (Figure 2A). We first constructed si-LINC00467 or pcDNA-LINC00467 and tested their transfection efficacies in glioma cells. We found that si-LINC00467 showed a better transfection efficacy in U87 cells (Figure 2B), and pcDNA-LINC00467 could markedly increase LINC00467 expression in LN229 cells (Figure 2C). After then, CCK8 and EdU assays were carried out to examine the effects of LINC00467 on cell proliferation capacity. As illustrated in Figure 2D-E, downregulation of LINC00467 significantly suppressed the growth of U87 cells, while upregulation of LINC00467 promoted cell proliferation of LN229 cells. To investigate the effects of LINC00467 on cell invasion, Matrigel assays were performed with treated cell lines. And the results disclosed that knockdown of LINC00467 reduced the invasion activity of U87 cells, however, upregulation of LINC00467 significantly increased the number of invasive LN229 cells (Figure 2F).

### LINC00467 promoted cell cycle and inhibited cell apoptosis

**Figure 3:**
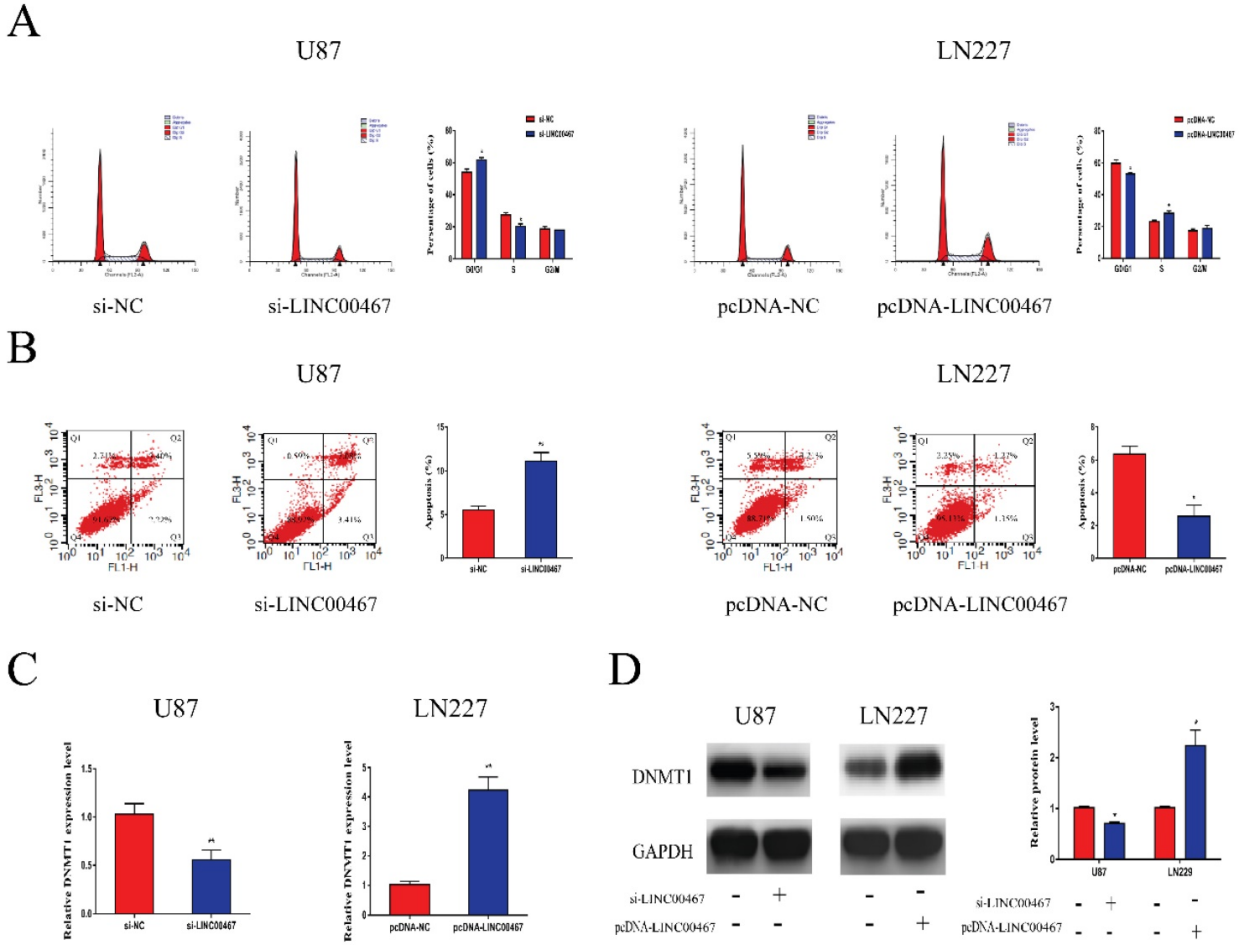
** LINC00467 promoted cell cycle and inhibited cell apoptosis** A. Flow cytometry was used to detect cell cycle. Knockdown of LINC00467 made cells arrested in G0/G1 phase while upregulation of LINC00467 promoted cell cycle. B. Flow cytometry was used to detect apoptosis rates. Knockdown of LINC00467 promoted cell apoptosis while upregulation of LINC00467 inhibited cell apoptosis. C and D. LINC00467 knockdown downregulated mRNA and protein expression of DNMT1 while overexpression of LINC00467 increased mRNA and protein expression of DNMT1 in glioma cells. The data represent the mean ± SEM from three independent experiments. *P < 0.05, **P < 0.01. mRNA, messenger RNA; DNMT1, DNA methyltransferase 1.

Flow cytometry was used to determine whether LINC00467 has an effect on cell cycle and apoptosis. As illustrated in Figure 3A, downregulation of LINC00467 increased the G0/G1 arrest in U87 cells, while LN229 cells treated with pcDNA-LINC00467 reduced the proportion of G0/G1 phase. In addition, we also observed that knockdown of LINC00467 in U87 can induce cell apoptosis, while upregulation of LINC00467 led to the opposite (Figure 3B). Previous study reported that LINC00467 might participate in cellular progress via positively regulating DNMT1 expression. Hence, we detected the expression of DNMT1 in glioma cells at both protein and mRNA levels. The results indicated that down-regulation of LINC00467 markedly reduced the expression of DNMT1; whilst up-regulation of LINC00467 increased the expression levels of DNMT1 (Figure 3C, 3D).

### LINC00467 inhibited p53 expression via binding to DNMT1

**Figure 4:**
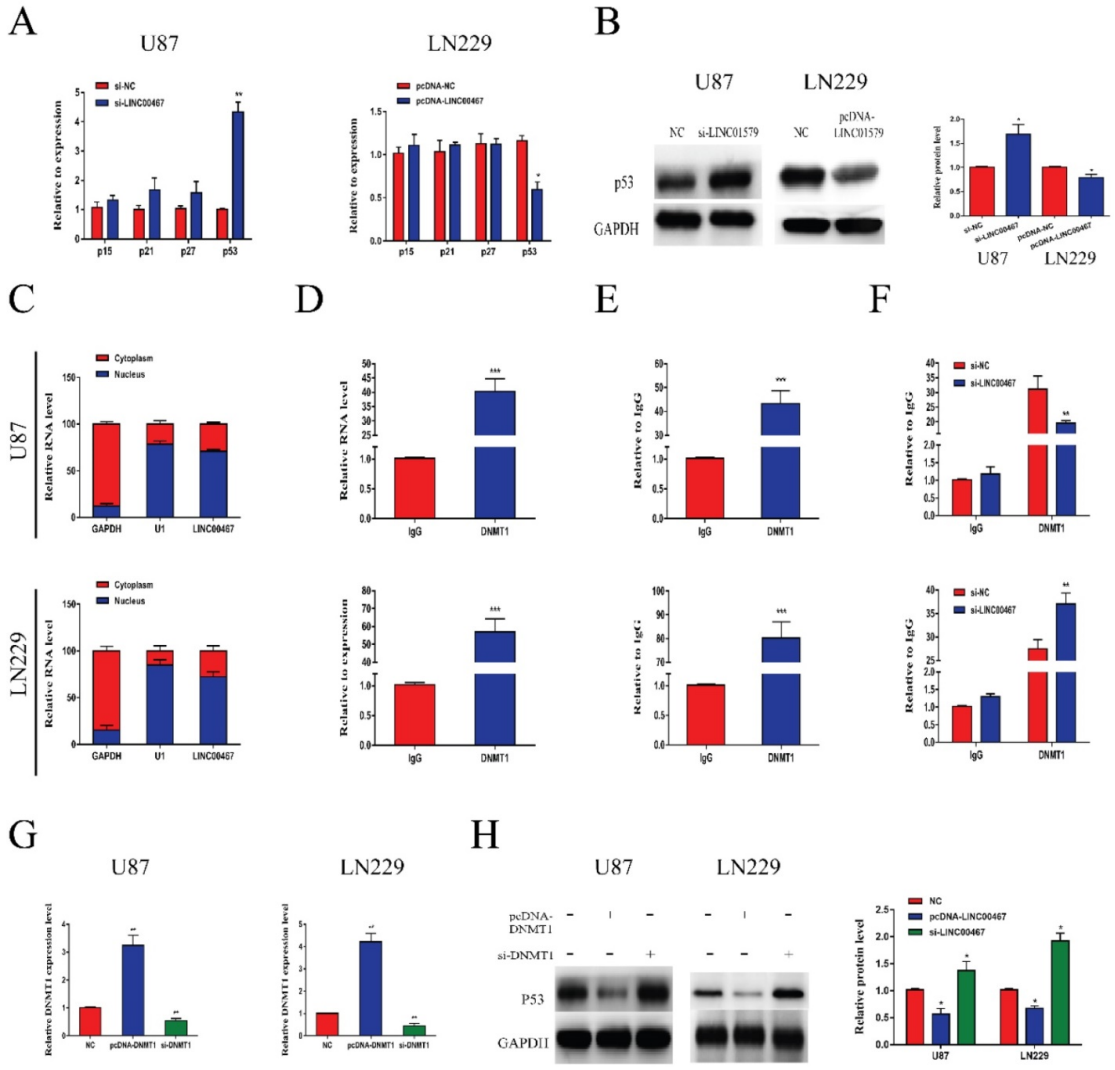
** LINC00467 inhibited p53 expression by binding to DNMT1** A and B. LINC00467 knockdown upregulated mRNA and protein expression of p53 in U87 cells, while overexpression of LINC00467 suppressed mRNA and protein expression of p53 in LN229 cells. C. Nuclear‐cytoplasmic separation assay showed that LINC00467 was mainly distributed in nuclear fractions of glioma cells. D. RIP results demonstrated that LINC00467 could be bound to DNMT1. E. ChIP results indicated that DNMT1 binds to DNAs in the p53 promoter region. F. Knockdown of LINC00467 in U87 cells downregulated the binding level of DNMT1 and p53 promoter, while overexpression of LINC00467 in LN229 cells upregulated the binding level of DNMT1 and p53 promoter. G. Transfection efficacy of si-LINC00467 and pcDNA-LINC00467 in glioma cells. H. After interfering with si-DNMT1 in U87 cells, p53 expression significantly increased. Upregulation of DNMT1 in LN229 cells, p53 expression markedly decreased. The data represent the mean ± SEM from three independent experiments. *P < 0.05, **P < 0.01, ***P < 0.001. mRNA, messenger RNA; DNMT1, DNA methyltransferase 1; RIP, RNA immunoprecipitation; CHIP, Chromatin immunoprecipitation.

To further explore the biological function of LINC00467, we detected the mRNA levels of oncogenes p15, p21, p27 and p53 in loss- and gain-of-LINC00467 glioma cell lines. The results of qRT-PCR showed that LINC00467 could negatively regulate p53 expression while had no obvious effect on other oncogenes at mRNA levels (Figure 4A). Similarly, Western blot results found that protein expression of p53 increased after transfection of si-LINC00467, and decreased after transfection of pcDNA-LINC00467 (Figure 4B). Moreover, we performed nuclear-cytoplasmic separation assay to determine the subcellular localization of LINC00467 in LN229 and U87 cells. As illustrated in Figure 4C, we found that LINC00467 was mainly distributed in nucleus of glioma cells, which indicated that LINC00467 might play its regulatory role at the transcriptional level (Figure 4C). Many studies proved DNMT1 can bind to p53 promoter region and inhibit its expression. We hypothesized that LINC00467 may suppress p53 expression via binding to DNMT1. Then we investigated the p53 promoter region encompassing the 2000 bp upstream of the p53 transcription start site and identified 6 candidate CpG islands in this region (Figure S1). Then we conducted RIP and ChIP assays to verify the combination relationship. The results of RIP assay elucidated that LINC00467 could bind to DNMT1 in U87 and LN229 cells (Figure 4D). The ChIP assay demonstrated that DNMT1 could bind to DNAs in the p53 promoter region in glioma cells (Figure 4E). What's more, the binding levels of DNMT1 and p53 promoter was decreased when si-LINC00467 were transfected into the cells and upregulated with pcDNA-LINC00467 interference (Figure 4F). After cells transfected with si-DNMT1, the mRNA expression levels of DNMT1 were markedly downregulated, while DNMT1 expression was increased when cells transfected with pcDNA-DNMT1 (Figure 4G). The results of western blot assay showed that DNMT1 can negatively regulate p53 protein expression (Figure 4H). All above results indicated that LINC00467 might inhibit p53 expression through binding to DNMT1.

### P53 reversed the effects of LINC00467 on cell proliferation and metastasis

**Figure 5:**
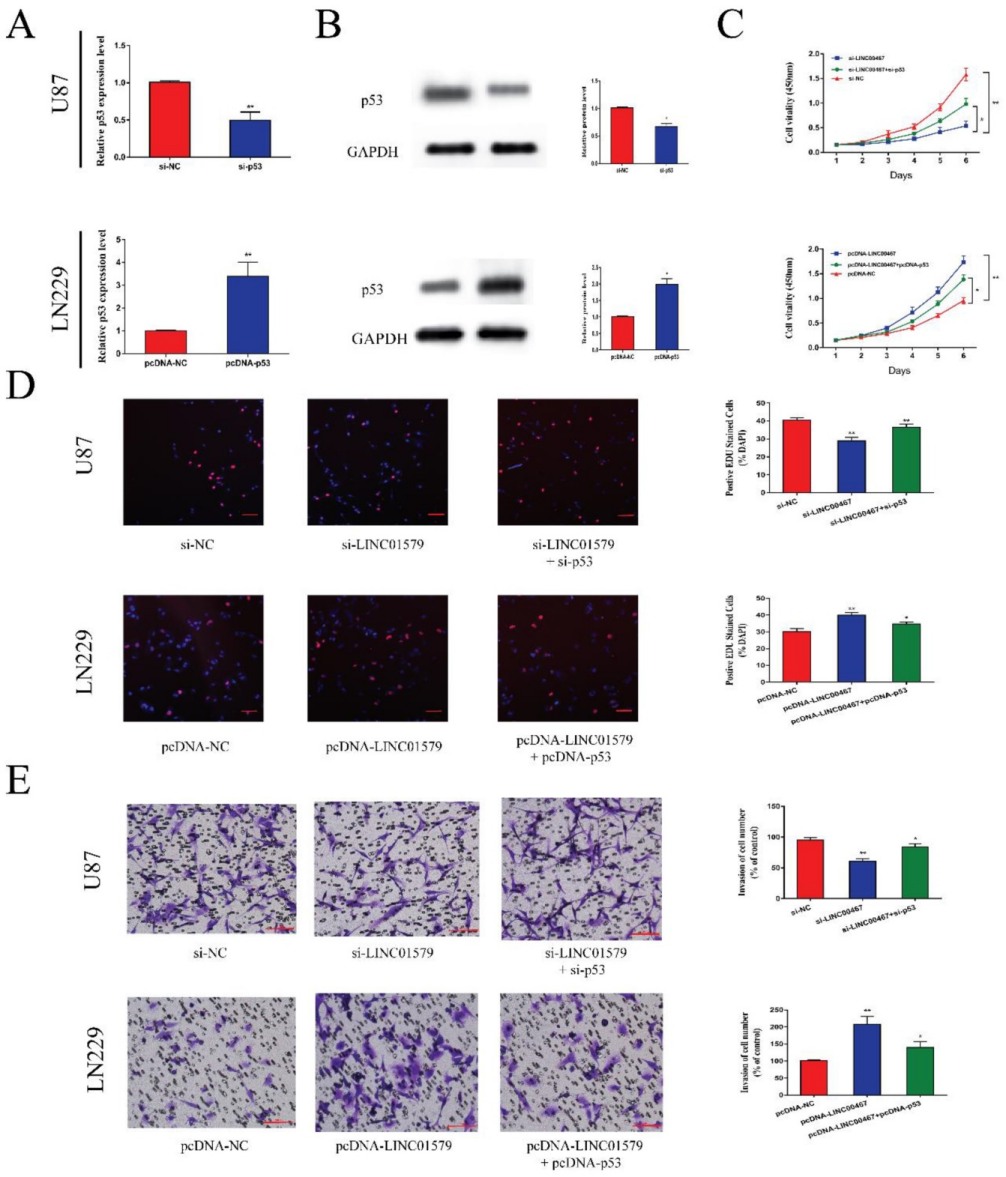
** P53 reversed the anti-tumor effect of LINC00467 on glioma** A, B. After transfection of si-p53 and pcDNA-p53 in glioma cells, mRNA and protein levels of p53 correspondingly changed. C and D. Cell proliferative ability was significantly decreased after downregulation of LINC00467, which was reversed by knockdown of p53. Similarly, cell proliferative ability could be enhanced after overexpression of LINC00467 and reversed by overexpression of p53. E. Cell invasive ability was significantly suppressed after knockdown of LINC00467, which was reversed by downregulation of p53. Moreover, cell invasive ability was significantly enhanced after overexpression of LINC00467, which was reversed by overexpression of p53. The data represent the mean ± SEM from three independent experiments. *P < 0.05, **P < 0.01. mRNA, messenger RNA.

We transfected glioma cells with si-p53 or pcDNA-p53 and performed qRT-PCR and Western blot assay to test the transfection efficiency. The results showed that si-p53 could reduce the mRNA and protein levels of p53, while pcDNA-p53 could upregulate the expression of p53 (Figure 5A and 5B). Then we performed CCK8 and EdU assays to detect cell proliferative ability. We found that cell proliferation was markedly attenuated after downregulation of LINC00467, which was partially reversed after co-transfected with si-p53 in U87 cells. Moreover, the ability of cell proliferation was significantly enhanced after overexpression of LINC00467, which was reversed by overexpression of p53 (Figure 5C, D). Similarly, the effect of LINC00467 on cell invasion ability could be reversed by p53 (Figure 5E). These findings indicated that LINC00467 could foster cell growth and invasion by inhibiting the expression of p53.

## Discussion

Malignant gliomas, with high risk of invasion and high mortality, are considered as the most common form of primary intrinsic brain tumors around the world 18, 19. LncRNAs are regarded as critical roles in the pathogenesis of glioma 20. Many lncRNAs have been identified to be dysregulated in glioma patients, which may contribute to glioma abnormal growth and metastasis. For instances, lncRNA metastasis-associated lung adenocarcinoma transcript 1 (lncMALAT1) acts as a tumor-suppressive role in glioma by downregulating matrix metallopeptidase 2 (MMP2) and inactivating the ERK/MAPK signaling 21, upregulation of lncHOXA-AS3 in glioma fosters tumor progression and indicates poor prognosis 22, lncRNA colon cancer‑associated transcript 2 (lncCCAT2) is highly expressed in glioma and can promote cell growth and migration 23. Given that current standard treatments, including surgery, radiotherapy and traditional chemotherapy, barely have any impact on the general prognosis of patients with glioma 24, 25, alternative therapeutic strategies are needed to be explored.

Previous studies have demonstrated that LINC00467 was upregulated in various types of malignant tumors, which contributed to tumorigenesis through different kinds of pathways. However, the functional roles and potential mechanism of LINC00467 in glioma tumorigenesis remain unknown. In this study, LINC00467 was found to be abnormally expressed in glioma through the detection database. Then we also discovered that LINC00467 is markedly upregulated in glioma cell lines compared with negative control cells (HEB). What's more, the results of cell function assays revealed that the upregulation of LINC00467 significantly promoted cell growth, invasion and accelerated cell cycle and inhibited cell apoptosis in glioma cells.

As is known to us, lncRNAs could regulate cellular biological progress at different levels including transcription, post-transcriptional modifycations and translation 26, 27. Hence, we performed experiments and found that LINC00467 mainly located in nucleus of glioma cells, which meant that LINC00467 might play its function at the transcriptional level. Wang et al., reported that LINC00467 contributes to lung adenocarcinoma cell proliferation and metastasis via binding with EZH2 and repressing HTRA3 expression 17. Accumulating evidence has demonstrated that lncRNAs could bind to transcription factors to take a part in target gene regulation 28. Then RIP assay was performed to verify the combination relationship between LINC00467 and DNMT1. The results of experiments elucidated that DNMT1 could bind to LINC00467, and LINC00467 was capable of stabilizing DNMT1 expression. DNMT1 is the primary enzyme that maintains the methylation pattern onto daughter strands after DNA replication 29. In many tumor cells, including glioma cells, high methylation of tumor-suppressor genes and abnormal cell proliferation and apoptosis are associated with increased DNMT1 activity 30. Previous studies proved that DNMT1 can bind to p53 promoter region and inhibit its expression in glioma 31, 32. Tumor suppression is as a cellular defense mechanism, tumor suppression can prevent normal cells from neoplastic transformation, and p53, a tumor suppressor, serves as an essential component in the initiation and progression of glioma 33. Hence, CHIP assay and western blot assay were performed to verify the binding condition between LINC00467, DNMT1 and p53. As expected, DNMT1 was able to bind to p53 and inhibited p53 expression, while LINC00467 could suppress p53 expression via binding to DNMT1. Then, CCK8 and EdU assays, transwell invasion assays were performed to verify the effect of LINC00467 on p53. All the results of experiments demonstrated that p53 could reverse the impact of LINC00467 on glioma cells, which meant that LINC00467 may promote cell proliferation and invasion by reducing p53 expression.

## Conclusions

In conclusion, this study revealed that high expression of LINC00467 can promote cell growth and invasion and inhibit the apoptosis of glioma cells, potentially via the inhibition of p53 expression by binding to DNMT1.

## Supplementary Material

Supplementary figure.

## Figures and Tables

**Table 1:** Sequences of primers for qRT-PCR

Name		Sequence
LINC00467	Forward	5'- GCCAGAGCAAGACTCTGTCTAC -3'
	Reverse	5'- GATGGGATACACATTCAATCAT -3'
p15	Forward	5'-GAAGAATCCAACAACGGC-3'
	Reverse	5'-TCACAATCAGGGAAGCAT-3'
GAPDH	Forward	5'-GCACCGTCAAGGCTGAGAAC-3'
	Reverse	5'-GGATCTCGCTCCTGGAAGATG-3'
p21	Forward	5'- CGACGCGTCGTTGTAATAAAGCCTCCAG -3'
	Reverse	5'- GACTAGTCGTTTTCAT TTCAATCGTAG -3'
p27	Forward	5'- TACTGGCACCACTGGAAACC -3'
	Reverse	5'-GACCACTGAGGTTAGAGCCA-3'
p53	Forward	5'- CTTGTGCCCTGTGAGGTCGTTGA -3'
	Reverse	5'-GACCACTGAGGTTAGAGCCA-3'
DNMT1	Forward	5'- TGGGAACTATATCTCTCGCTTGC -3'
	Reverse	5'- GGGTGAGACAGAACCGTCT -3'
U6	Forward	5'- GCTTCGGCAGCACATATACTAAAAT-3'
	Reverse	5'- CGCTTCACGAATTTGCGTGTCAT -3'
