# Supplementary material for: Long Noncoding RNA LINC00467 Promotes Glioma Progression through Inhibiting P53 Expression via Binding to DNMT1
Source: J Cancer. 2020 Mar 4;11(10):2935–44. doi: 10.7150/jca.41942 (PMC7086258; doi:10.7150/jca.41942)
Supplement: Supplementary file 1 — Supplementary figure. [file jcav11p2935s1.pdf]

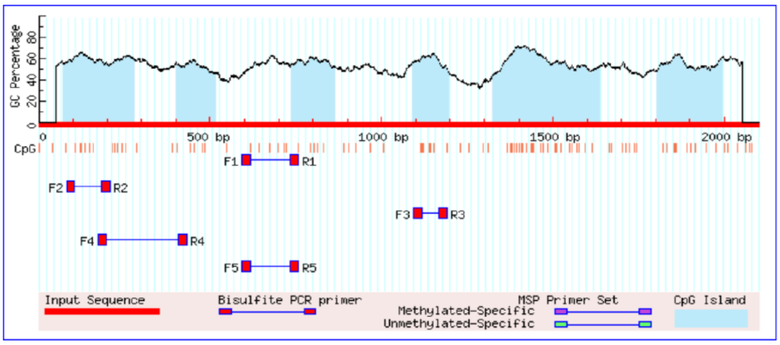

CpG island prediction results
(Criteria used: Island size > 100, GC Percent > 50.0, Obs/Exp > 0.6)
6 CpG island(s) were found in your sequence

	Size	(Start - End)
Island 1	208 bp	(71 - 278)
Island 2	116 bp	(402 - 517)
Island 3	127 bp	(736 - 862)
Island 4	107 bp	(1089 - 1195)
Island 5	313 bp	(1324 - 1636)
Island 6	191 bp	(1804 - 1994)

Figure S1. Schematic diagram of the p53 promoter region, referring to http://www.urogene.org/methprimer/. The p53 promoter contains 6 candidate CpG islands.
